# CAISHI: A benchmark histopathological H&E image dataset for cervical adenocarcinoma *in situ* identification, retrieval and few-shot learning evaluation

**DOI:** 10.1016/j.dib.2024.110141

**Published:** 2024-02-09

**Authors:** Xinyi Yang, Chen Li, Ruilin He, Jinzhu Yang, Hongzan Sun, Tao Jiang, Marcin Grzegorzek, Xiaohan Li, Chang Liu

**Affiliations:** a Microscopic Image and Medical Image Analysis Group, College of Medicine and Biological Information Engineering, Northeastern University, Shenyang, Liaoning 110167, China; b Key Laboratory of Intelligent Computing in Medical Image, Ministry of Education, Northeastern University, Shenyang, Liaoning 110167, China; c Shengjing Hospital of China Medical University, Shenyang, Liaoning 110001, China; d School of Intelligent Medicine, Chengdu University of Traditional Chinese Medicine, Chengdu, Sichuan 610075, China; e International Joint Institute of Robotics and Intelligent Systems, Chengdu University of Information Technology, Chengdu, Sichuan 610225, China; f Institute for Medical Informatics, University of Luebeck Ratzeburger Allee, Luebeck 160 23538, Federal Repulic of Germany; g Department of Knowledge Engineering, University of Economics in Katowice, Katowice 50 40-287, Poland

**Keywords:** Cervical cancer, Image classification, Deep learning, Machine learning, Computer vision

## Abstract

A benchmark histopathological Hematoxylin and Eosin (H&E) image dataset for Cervical Adenocarcinoma *in Situ* (CAISHI), containing 2240 histopathological images of Cervical Adenocarcinoma *in Situ* (AIS), is established to fill the current data gap, of which 1010 are images of normal cervical glands and another 1230 are images of cervical AIS. The sampling method is endoscope biopsy. Pathological sections are obtained by H&E staining from Shengjing Hospital, China Medical University. These images have a magnification of 100 and are captured by the Axio Scope. A1 microscope. The size of the image is 3840 × 2160 pixels, and the format is “.png”. The collection of CAISHI is subject to an ethical review by China Medical University with approval number 2022PS841K.

These images are analyzed at multiple levels, including classification tasks and image retrieval tasks. A variety of computer vision and machine learning methods are used to evaluate the performance of the data. For classification tasks, a variety of classical machine learning classifiers such as *k*-means, support vector machines (SVM), and random forests (RF), as well as convolutional neural network classifiers such as Residual Network 50 (ResNet50), Vision Transformer (ViT), Inception version 3 (Inception-V3), and Visual Geometry Group Network 16 (VGG-16), are used. In addition, the Siamese network is used to evaluate few-shot learning tasks. In terms of image retrieval functions, color features, texture features, and deep learning features are extracted, and their performances are tested. CAISHI can help with the early diagnosis and screening of cervical cancer. Researchers can use this dataset to develop new computer-aided diagnostic tools that could improve the accuracy and efficiency of cervical cancer screening and advance the development of automated diagnostic algorithms.

Specifications Table

**Table:** 

Subject	Health and medical sciences Biomedical Engineering
Specific subject area	Cross-disciplinary healthcare solutions (defined as the application of engineering principles and design concepts to medicine and biology for healthcare purposes, including diagnostic or therapeutic). Similar terms: bioengineering, medical engineering.
Data format	Filtered data in both .png (Number and name the data. The corresponding record file is .xlsx) formats. (Note: For data balance, 1010 images of normal and cervical AIS are mainly used.)
Type of data	.png file (dataset with images) .xlsx file (dataset with labels)
Data collection	Images of CAISHI are obtained by endoscopic biopsy sampling method, and then pathological sections are made by H&E staining at Shengjing Hospital of China Medical University. The images, with a magnification of 100, are taken by the Axio Scope.A1 microscope. The size of the image is 3840 × 2160 pixels and the format is “.png”.
Data source location	Shengjing Hospital of China Medical University, Shenyang, Liaoning, China
Data accessibility	The collection of the CAISHI dataset is subject to an ethical review by China Medical University with approval number 2022PS841K. Repository name: MIaMIA-Open-Data-Cervical-AIS-Histopathology-Image All data can be accessed at the following link: https://doi.org/10.6084/m9.figshare.24548953

## 1 Value of the Data

• Currently, there is a lack of publicly available histopathological image datasets for cervical AIS, and CAISHI is one such dataset.
• This data can benefit doctors, pathologists, biomedical engineers, computer vision and machine learning researchers, among others. They can use this data to improve the accuracy and efficiency of early diagnosis and screening of cervical cancer, thereby reducing the incidence and mortality of cervical cancer.
• This data can be reused by other researchers in a number of ways, such as different pre-processing and enhancement of the data to improve image quality and variety; Different feature extraction and classification methods are used to compare the performance of different algorithms in cervical AIS recognition. Use the data in combination with other relevant datasets to expand the sample size and cover more cervical cancer types; Transfer learning or meta-learning of deep learning models using data to improve the generalization ability and robustness of the models.

## 2 Background

Cervical cancer has a high incidence worldwide and is the fourth most common cancer among women [1]. Cervical AIS is considered to be the true precursor of adenocarcinoma [2]. Histopathology is the study of disease through tissue sections. In Ref. [3], Li et al. classified more than 600 immunohistochemical (IHC) color samples and 200 H&E stained cervical histopahological datasets, with the highest accuracy of 88% for the former and 93% for the latter. In Ref. [4], Xue et al. proposed an integrated transfer learning framework for classification on a dataset of 307 cervical histopathological images (stained by AQP, HIF, and VEGF). In Ref. [5], Wang et al. presented a histopathological full slide image dataset for classification of ovarian cancer treatment efficacy, consisting of 288 de-identified H&E stained WSI (including 162 valid WSI and 126 ineffective WSI). All data set information is summarized in Table 1.

**Table 1:** Recent datasets of cervical cancer.

Year	Name	Ref.	Categorization	Amount
2019	Six practical cervical and a gastric histopathological image datasets	[3]	IHC and H&E stained cervical histopathological images	600; 200
2020	A cervical histopathological images	[4]	IHC (AQP, HIF, AND VEGF) cervical histopathological images	307
2022	BreCaHAD	[5]	Contains H&E stained breast cancer histopathological images	162

At present, there is a lack of published pathological image datasets of cervical AIS as the gold standard for diagnosis, and the published images of cervical cancer datasets are not many. The goal of establishing the dataset is to address the gap in the availability of the dataset.

## 3 Data Description

The Cervical AIS dataset is collected from 52 patients with cervical AIS at Shengjing Hospital of China Medical University, with 1–67 slices per case, depending on the number of tissue sizes. 60 patients with normal cervix are collected, and 1–69 tablets are taken for each case according to the number of different tissue sizes. The final CAISHI dataset consists of 2240 images, including 1230 cervical AIS histopathology images and 1010 normal cervical images. Our dataset is an extension of the previous dataset [6]. The images, with a magnification of 100, are taken by the Axio Scope.A1 microscope and the size of the image is 3840 × 2160 pixels.

- data.xlsx: A meticulously compiled comparison table, correlating hospital-given names with manually assigned numerical identifiers for each image.
- Abnormal: A dedicated folder containing 1230 AIS histological images of the cervix. Each image in this folder is named according to a specific convention, such as “Abnormal-0001” for easy identification.
- Normal: This folder houses 1010 images depicting normal cervical glands. Similar to the Abnormal folder, images here are systematically named for straightforward reference.

**Fig. 1:**
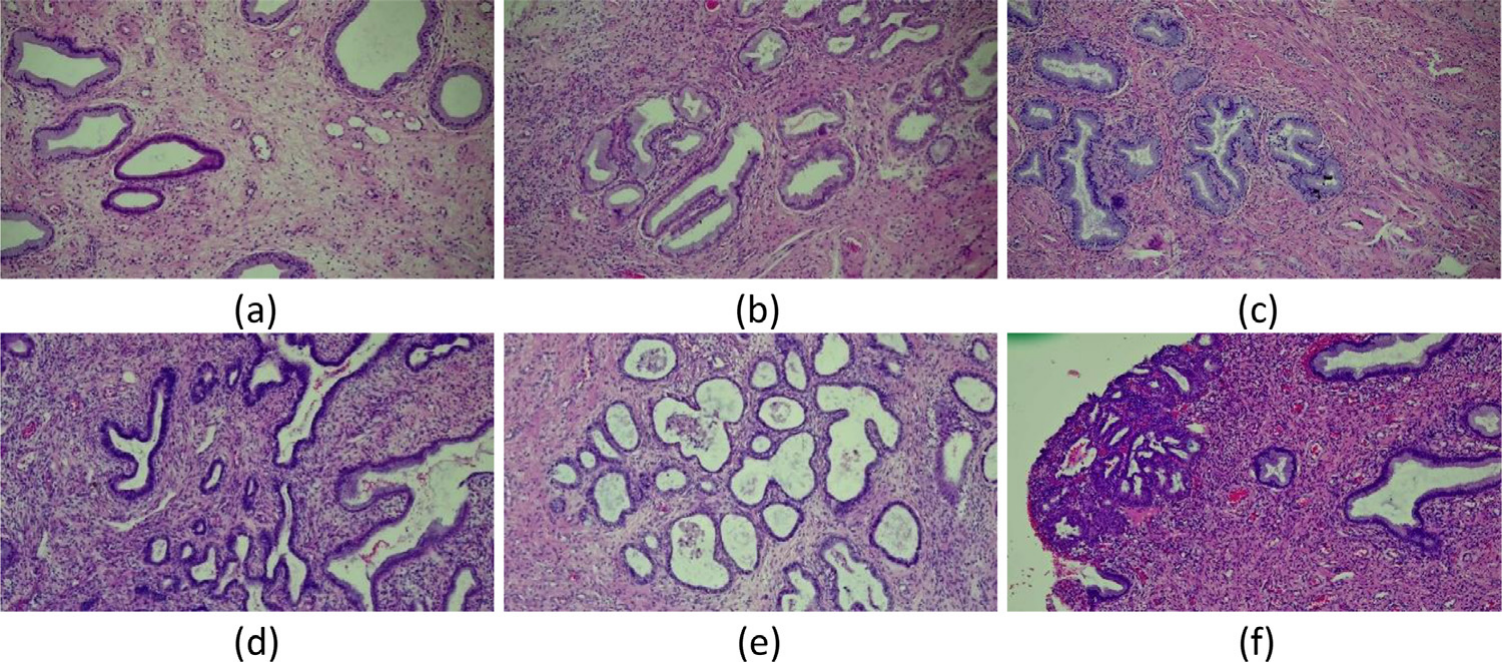
An example of CAISHI (H&E, 100×): (a) (b) and (c) Normal images, (d) (e) and (f) Abnormal images.

Examples of images in the dataset are shown in Fig. 1(a)–(c) are images of normal cervical glands, (d)–(f) are images of cervical AIS.

Normal images do not contain areas of cancer, and each cell either has no or very little heterotypy. Additionally, the nuclei in the image are well-organized and nearly free of mitosis (Fig. 1(a)–(c)). An image can be considered normal if no loss of cells or tissues is observed when it is examined under a light microscope and the conditions for a normal picture are met.

In the image of cervical AIS, the structure of cervical mucosal glands is preserved, but some epithelial cells on the surface of the cervical endometrium or glands change. This change is characterized by nuclear enlargement, chromatin thickening, single or multiple nucleoli, increased nuclear division activity, and varying degrees of nuclear stratification (Fig. 1(d)–(f)). The mucus in the cytoplasm of the affected epithelial cells is reduced, and sometimes papillary structures can appear in the glandular cavity [7]. Seeing mitotic figures in the diseased glandular epithelium, especially on the surface of the glandular cavity, can be used as an important basis for distinguishing benign glandular epithelial lesions [8].

The staining procedure is commonly regarded as the gold standard in H&E paraffin slice technology. Cell nuclei are stained purple-blue by hematoxylin, whereas the extracellular matrix and cytoplasm are stained pink by eosin, an acidic dye. Other structures appear in different tints and shades of these colors [9]. This staining pattern gives pathologists a basic understanding of the structure of the tissue and cell distribution in addition to making it simple to distinguish the nuclear and cytoplasmic parts of cells. The pink and white regions are more prominent in the pathological image following H&E staining, but the purplish blue area is more dispersed and disorderly in the abnormal image. Cervical AIS diagnostic criteria: nuclear enlargement, hyperchromatism, and atypia, visible nuclear upshift, nuclear division, or increased number of apoptotic bodies, etc.

## 4 Experimental Design, Materials and Methods

The CAISHI dataset forms the basis of our experiment, focused on categorizing images into two classes: normal cervical glands and cervical AIS. The Cervical AIS dataset is collected from 52 patients with cervical AIS at Shengjing Hospital of China Medical University, with 1–67 slices per case, depending on the number of tissue sizes. 60 patients with normal cervix are collected, and 1-69 tablets are taken for each case according to the number of different tissue sizes and the final CAISHI dataset consists of 2240 images, including 1230 cervical AIS histopathology images and 1010 normal cervical images. The test methods include various image processing techniques, employing both traditional machine learning and advanced deep learning methodologies. This section details the approaches used in feature extraction, classification, retrieval, and evaluation.

### 4.1 Experimental Setup

The experiments are conducted on a laptop equipped with 32GB RAM, Windows 10 OS, and a GeForce RTX 2060 GPU from NVIDIA. The Matlab programming language is used for traditional machine learning techniques, while Python 3.9 and Pytorch 1.10.1 are utilized for deep learning methods.

### 4.2 Feature Extraction

Five key characteristics are extracted for classification and retrieval. Color Histogram is extracted by evaluating the distribution of colors within the image, focusing on the HSV histogram (Hue, Saturation, Value) color space to align with human visual perception. Texture Features including Histogram of Oriented Gradients (HOG), Local Binary Patterns (LBP), and Gray-level Co-occurrence Matrix (GLCM), are extracted using MATLAB. Deep Learning Features are extracted from the last layers of ResNet50, Vision Transformer (ViT), Inception-V3, and VGG-16 models implemented in PyTorch.

### 4.3 Image Classification

The aim of this experiment is to classify the CAISHI dataset into two categories: normal cervical glands and cervical AIS. Following feature extraction, the CAISHI dataset is classified using traditional machine learning techniques including SVM, RF, and *k*-means. In addition, four popular and new deep learning methods are also used for classification, including ResNet50, ViT, Inception-V3, and VGG-16. The evaluation metrics used in this experiment are accuracy, precision, recall, and F1-score. The same five-fold cross-validation is performed using these seven classifiers and rotated using 1 fold for testing and 4 folds for training. The number of trees in the RF is set to 10. A linear kernel is used for the kernel function of the SVM. Besides, 30 epochs are conducted to identify the impacts of various models on CAISHI with a learning rate of 0.0001 for each model and a batch size of 8.

For few-shot learning, a large training dataset Mini-ImageNet is used to train the Siamese Network [10]. The Siamese network has two identical convolutional neural networks, which compute feature vectors for their input images and then compare the similarity of the images, i.e., the similarity relationship between different images is mapped to the metric space. Here the Triplet Loss [11] proposed by Google in the 2015 FaceNet paper is used to train the twin network. For each training, three images are selected from the training set: first, select a random image from the dataset and use it as an anchor; second, select another image randomly in the same category as a positive sample; and finally, select a random image in a different category and use it as a negative sample. Feature extraction is performed using ResNet50 based on transfer learning. The three images are fed into the convolutional neural network. After that, the features are extracted to get three feature vectors, and then the square of the Euclidean distance between the positive sample feature vector and the anchor feature vector is calculated. At the same time, the same calculation is performed for the negative sample feature vector. The former distance is expected to be large and the latter distance is small, and the former is much larger than the latter to define the loss function so that features with the same label can be organized as close as possible to each other in space, while features with different labels are separated in space.

After completing the training, the model is subjected to prediction. The first 1010 images of each category of the CAISHI dataset are taken and divided into 10 sets. Here a 2-way 1-shot support set is used, where each support set consists of one normal cervical gland and one cervical AIS image, and the query consists of 100 normal cervical glands and 100 cervical AIS images. The model has not seen the sample category of cervical AIS, so let the model determine whether the currently given query image belongs to the normal cervical gland or cervical AIS in the support set. For example, the query is compared with the sample in the Support Set, and the category with the greatest similarity or the least distance is output.

The same parameters are used in all classification experiments. SVM using a linear kernel function, RF with 10 trees, and *k*=2 for *k*-means. As can be seen from Table 2, the best performing feature in CAISHI is the color feature, which gives good classification results in both linear SVM and RF. The best classification result is RF using color features, with 90.18% classification accuracy, precision, recall, and F1-score above 90%. However, RF is not as good as linear SVM when using other features for classification. Linear SVM also has good classification results when using GLCM, LBP, and HOG features for classification. The accuracy, precision, recall, and F1-score are higher than those of other classifiers. In comparison, *k*-means classification is less effective.

**Table 2:** Classification results of CAISHI using different classifiers for four image features (In [%]).

Features	Methods	Acc	Abnormal			Normal		
			Precision	Recall	F1-score	Precision	Recall	F1-score
Color Histogram	Linear							
	SVM	77.14	83.75	72.68	77.72	71.37	82.57	76.48
	RF	90.18	92.28	92.04	91.12	90.22	87.92	89.01
	*k*-means	46.12	50.75	63.33	56.35	36.03	25.15	29.62
HOG	Linear							
	SVM	64.37	67.29	69.43	68.32	61.46	59.01	60.18
	RF	56.79	58.54	73.17	65.03	53.55	36.83	43.39
	*k*-means	53.13	57.76	54.47	56.07	48.15	51.49	49.76
LBP	Linear							
	SVM	71.10	74.51	73.34	74.06	68.54	69.21	68.79
	RF	60.58	61.49	75.53	67.78	58.75	42.37	49.20
	*k*-means	44.33	49.55	76.67	60.20	14.84	4.95	7.42
GLCM	Linear							
	SVM	74.38	75.57	78.86	77.18	72.78	68.91	70.79
	RF	69.91	70.87	76.75	73.68	68.54	61.59	64.87
	*k*-means	58.88	59.72	77.15	67.33	56.84	36.63	44.55

Among the four neural network classifiers, the classification results are good. As shown in Table 3. Almost all of them are above 90%, with ResNet50 classification accuracy reaching 96.88% and Inception-V3 reaching 96.70%. In few-shot learning, the dataset performs quite well with an accuracy rate of around 75%, as shown in Table 4.

**Table 3:** Classification results of four deep learning dassifiers on CAISHI (In [%]).

Model	Acc	Category	Precision	Recall	F1-score
ResNet50	96.88	Abnormal Normal	97.15 96.15	96.67 96.45	96.58 95.62
ViT	89.96	Abnormal Normal	90.14 89.43	91.72 86.97	89.79 86.29
Inception-V3	96.70	Abnormal Normal	97.58 95.52	96.41 97.00	96.65 95.68
VGG-16	92.59	Abnormal Normal	92.89 91.55	94.00 89.99	92.31 89.25

**Table 4:** Few-shot learning results (In [%]).

Model	Backbone	Category	1-shot
Siamese Network	ResNet50	Abnormal Normal	78.4 75.2

### 4.4 Image Retrieval

In this work, CAISHI is utilized for image retrieval, which, depending on the features used, may be separated into texture feature-based and deep learning feature-based methodologies. Average Precision (AP) [12], a statistic frequently used in information retrieval to assess the performance of ranked lists of retrieved samples, is utilized to assess the efficacy of retrieval techniques. Specifically, AP is defined as shown in Eq. (1).

(1) $$\text{AP}=\frac{\sum_{i=1}^{n}\left(P\left(i\right)\times \text{rel}\left(i\right)\right)}{N}\;$$

In the equation, *N* represents the number of related images, P(i) is the precision value at the *i*th position in the list when considering the cut-off position, and rel(i) is an index that indicates whether the image in the *i*th position of the list is the target type image. If the image in the *i*th position is the target type image, rel(i) is set to 1; otherwise, it is set to 0. AP is the average value of the precision at each position where the target type image is found. As the experiment is conducted on two types of images, the mean average precision (mAP) is calculated by averaging the APs of each class.

Eight feature vectors in total are employed, including the final layer of the ResNet50, ViT, Inception-V3, and VGG-16 networks as well as four previously extracted features, color features, HOG features, LBP features, and GLCM features. The feature vectors of the picture are evaluated against the feature vectors of every image in the CAISHI dataset throughout the retrieval process, and the Euclidean distance between them is determined. The mAP value is then determined for the test picture type. The search result for the test kind of picture is then computed to provide the mAP value. The first 20 images are shown in one set of results, while all of the retrieved results are shown in the other.

**Fig. 2:**
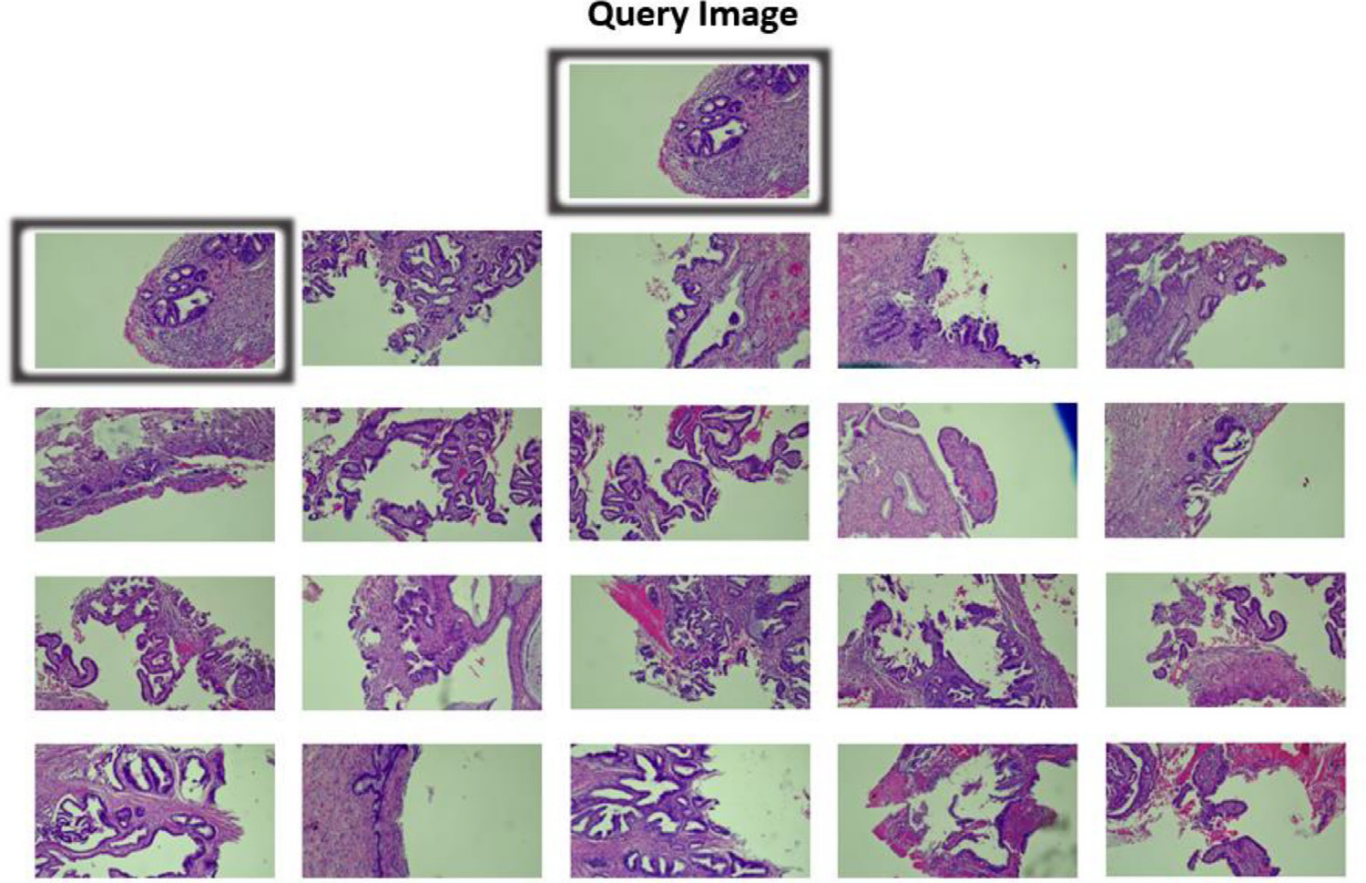
An example of image retrieval results based on color feature using CAISHI.

An image retrieval effect is schematically shown in Fig. 2.

To evaluate the image retrieval evaluation function of CAISHI, the color feature, HOG, LBP, GLCM, and the final layer of feature vectors of the four neural networks are retrieved. Table 5 shows the accuracy evaluation of their image retrieval results. Both color features and GLCM perform better in texture features, and color features can achieve the highest mAP@20 of 93.62% for cervical AIS. However, the results of picture retrieval using deep learning features are more reliable and accurate, and the mAP or mAP@20 could virtually approach over 90% for both normal cervical gland and cervical AIS retrieval. CAISHI images may be used efficiently for testing and evaluating various image retrieval algorithms by contrasting the outcomes of different retrieval techniques.

**Table 5:** mAP results of image retrieval (In [%]).

Feature	Category	mAP	mAP@20
VGG-16	Abnormal Normal	84.86 91.34	95.42 94.60
ResNet50	Abnormal Normal	93.58 97.95	98.96 98.95
Inception-V3	Abnormal Normal	98.09 96.82	99.40 99.35
ViT	Abnormal Normal	85.42 82.03	92.34 90.93
Color Histogram	Abnormal Normal	65.09 52.93	93.62 86.77
HOG	Abnormal Normal	42.07 60.47	65.47 71.27
LBP	Abnormal Normal	49.37 63.19	76.96 84.35
GLCM	Abnormal Normal	61.47 50.61	86.55 74.41

## Limitations

The dataset in this paper is based on cervical AIS stained by H&E from Shengjing Hospital of China Medical University, and may not be representative of the characteristics and distribution of cervical AIS in other regions or countries. It only includes two types of labels: normal cervical glands and cervical AIS, and do not consider other possible cervical lesions, such as cervical squamous cell carcinoma, cervical adenocarcinoma, and cervical intraepithelial neoplasia.

Images in the dataset are in “.png” format, which may have some degree of compression and distortion, affecting image quality and feature extraction.

## Ethics Statement

The collection of CAISHI is subject to an ethical review by China Medical University with approval number 2022PS841K.

## CRediT authorship contribution statement

**Xinyi Yang:** Methodology, Software, Formal analysis, Visualization, Writing – original draft, Writing – review & editing. **Chen Li:** Writing – review & editing, Supervision, Funding acquisition. **Ruilin He:** Methodology. **Jinzhu Yang:** Methodology. **Hongzan Sun:** . **Tao Jiang:** Methodology. **Marcin Grzegorzek:** Methodology. **Xiaohan Li:** Data curation. **Chang Liu:** Methodology.

## Data Availability

CASIHI (Original data).

## Appendix Supplementary materials

Image, application 1
