# Supplementary figures and images for: CAISHI: A benchmark histopathological H&E image dataset for cervical adenocarcinoma in situ identification, retrieval and few-shot learning evaluation
Source: Data Brief. 2024 Feb 9;53:110141. doi: 10.1016/j.dib.2024.110141 (PMC10885606; doi:10.1016/j.dib.2024.110141)

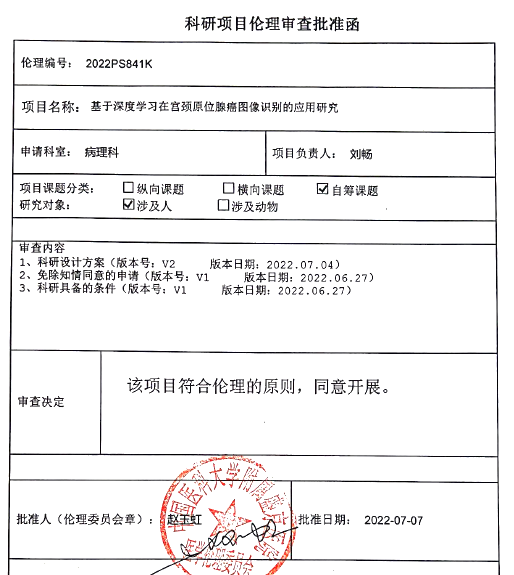

Supplement: Supplementary file 1 [file mmc1.docx]
